# Deficits in Performance on a Mechanically Coupled Asymmetrical Bilateral Task in Chronic Stroke Survivors with Mild Unilateral Paresis

**DOI:** 10.3390/sym13081366

**Published:** 2021-07-27

**Authors:** Shanie A. L. Jayasinghe, Candice Maenza, David C. Good, Robert L. Sainburg

**Affiliations:** 1 Department of Neurology, Pennsylvania State University College of Medicine, Hershey, PA 17033, USA; 2 Department of Kinesiology, Pennsylvania State University, State College, PA 16802, USA

**Keywords:** lateralization, bimanual, motor control, bilateral coordination, stabilizing

## Abstract

Typical upper limb-mediated activities of daily living involve coordination of both arms, often requiring distributed contributions to mechanically coupled tasks, such as stabilizing a loaf of bread with one hand while slicing with the other. We sought to examine whether mild paresis in one arm results in deficits in performance on a bilateral mechanically coupled task. We designed a virtual reality-based task requiring one hand to stabilize against a spring load that varies with displacement of the other arm. We recruited 15 chronic stroke survivors with mild hemiparesis and 7 age-matched neurologically intact adults. We found that stroke survivors produced less linear reaching movements and larger initial direction errors compared to controls (*p* < 0.05), and that contralesional hand performance was less linear than that of ipsilesional hand. We found a hand × group interaction (*p* < 0.05) for peak acceleration of the stabilizing hand, such that the dominant right hand of controls stabilized less effectively than the nondominant left hand while stroke survivors showed no differences between the hands. Our results indicate that chronic stroke survivors with mild hemiparesis show significant deficits in reaching aspects of bilateral coordination, but no deficits in stabilizing against a movement-dependent spring load in this task.

## 1. Introduction

Most activities of daily living (ADL) involving the upper extremity require coordinated interaction between the arms, such as when buttoning a shirt, cutting a slice of bread, or opening a jar. The range of ADL that require bilateral coordination suggests that bilateral coordination assessment and training should be an important focus of rehabilitation for stroke survivors who are able to use both arms for manipulation (high-moderate to mild impairment). Individuals with severe paresis are likely to be able to use the contralesional arm only for gross assistance purposes, if at all. Previous research has indicated that bilateral movement training can be a useful tool to promote functional recovery in stroke survivors [1–3]. Different mechanisms of action of bilateral training have been hypothesized [4], with a focus on the idea that spatiotemporal coupling of the two arms can facilitate recovery of motor function by recruiting specialized neural circuitry [5]. Bilateral training can increase activation of the ipsilesional primary motor cortex [6], and can also result in larger gains in stroke-related recovery of arm function than unilateral training of the affected arm alone [7,8]. Although both unilateral and bilateral training of upper-limb reaching tasks can improve paretic arm function [9], bilateral training was found to be more beneficial for improving proximal, but not distal, arm function related to bilateral descending motor pathways that support proximal limb function [10]. Unilateral training has been shown to improve paretic arm performance [11], but it may not provide the range of movement experiences required by functional activities [12] even though the therapeutic effects of bilateral training on stroke recovery remain incompletely understood [13].

Bilateral upper limb tasks must integrate the action of two effectors that are asymmetrical in terms of motor performance, a phenomenon that appears to emerge from the specialized contribution of each hemisphere to the contralateral arm [14,15]. Specifically, it has been shown that left and right hemisphere damage produces different motor deficits in both arms of premorbidly right-handed stroke survivors [16]. While left hemisphere damage tends to produce deficits in trajectory features, such as movement smoothness, linearity, direction specification, and initial acceleration features [17,18], right hemisphere damage produces deficits in movement deceleration [18,19] and in the ability to achieve an accurate and stable final position [20]. These studies, as well as studies in neurologically intact typical adults, have led to the hypothesis that the left hemisphere in right-handers is specialized for control of movement trajectory and intersegmental coordination, while the right hemisphere is specialized for the control of limb steady-state positions and control of limb impedance [21]. A large number of previous studies in typical adults and in unilateral stroke survivors have provided support for this model through the study of unilateral reaching movements [20,22].

It is thus reasonable to expect similar lateralized motor deficits to emerge during bimanual movements. Recently, a virtual bilateral coordination task with unilateral stroke survivors showed that damage to the left, not right, hemisphere produced deficits in trajectory control [23]. However, there were no differences between left and right hemisphere damage for achieving stable final positions for either hand, which may have had to do with the nature of the task that did not emphasize aspects of control that are generally deficient in individuals with right hemisphere damage (e.g., final position accuracy under no-vision conditions). In addition, the task did not include mechanical coupling between the arms, and thus was not an assessment of bilateral coordination under realistic conditions that might better reflect ADL. Our laboratory previously described bilateral coordination during a mechanically coupled bilateral task (involving an outward reaching movement with one hand while stabilizing against spring forces with the other hand) [24]. We reported that, in right hand-dominant neurologically intact individuals, the right hand made straighter reaching movements against the movement-dependent spring load than the left hand, while the left hand had greater positional stability against the spring load compared to the right hand. This supported the premise that previously identified hemispheric specializations for unilateral movements are also reflected in this mechanically coupled bilateral task.

We now exploit this paradigm to examine potential differences between neurologically intact typical adults (controls) and chronic stroke survivors with unilateral brain damage on a mechanically coupled asymmetrical bilateral task that requires an out-and-back reaching movement of one hand and maintenance of a steady position with the other hand. The task was designed to reflect the coordination requirements of ADL tasks because it requires control over the mechanical interactions between hands when stabilizing with one hand and moving with the other, similar to slicing bread or opening a jar. Hence, this task required a systematic coordination of the hands to compensate for the forces exerted by each on the other via a connecting spring. Since fairly skilled use of both hands is necessary for completing this bilateral task, we recruited stroke survivors with mild hemiparesis. We hypothesized that unilateral stroke survivors would exhibit greater deficits in control of both movement and posture compared to controls. We predicted that performance deficits in the contralesional hand would be larger than those in the ipsilesional hand based on previous studies that compared movement deficits between hands during unilateral reaching tasks [25,26]. Our results revealed deficits in reaching movements that were greater in stroke survivors compared to controls, especially in the contralesional hand. However, stroke survivors showed no differences in stabilizing performance between the two hands or when compared to performance of controls. These findings suggest that bilateral training is feasible in stroke survivors with mild paresis, and that remediation should focus on the movement, rather than the stabilization aspects of coordination.

## 2. Materials and Methods

We recruited 15 premorbidly right-hand-dominant unilateral chronic stroke survivors (Table 1) with mild contralesional arm deficits (4 females; mean age 61.67 years ± 3.11 SEM). Contralesional arm function was evaluated using the upper-extremity portion of the Fugl-Meyer assessment, which provides a score out of 66 total points [27]. All participants had a score between 49 and 66, which was defined as mild paresis according to a cluster analysis of the Fugl-Meyer [28]. Exclusion criteria for stroke survivors were bilateral lesions, a diagnosed cognitive disorder reported by neuropsychological tests or neurology clinical records and inability to follow instructions adequately to perform our clinical measures and participate in the experimental tasks, and non-stroke-related neurological diseases or upper extremity-related orthopedic dysfunction. A neurologist confirmed the presence of unilateral lesions by examining medical records and MRI scans of all stroke survivors. We used the Jebsen-Taylor hand function test [29], which involves seven timed coordination and manipulation tasks, to measure ipsilesional arm motor performance. We calculated the total time to complete each task except for the writing portion as premorbid hand dominance influences writing performance. Total times (without writing) above approximately 45 s were indicative of deficits in ipsilesional arm motor performance. We also recruited a control group of 7 neurologically intact right-hand dominant individuals (3 females; mean age 56.14 years ± 4.43 SEM). Exclusion criteria for the neurologically intact individuals included any current or prior upper extremity motor disorder or stroke, and any cognitive disorders. The Edinburgh Handedness Inventory was provided to confirm hand dominance [30]. We received written informed consent from all participants prior to study initiation, and all study procedures were approved by the Pennsylvania State University College of Medicine’s Institutional Review Board.

**Figure 1.**
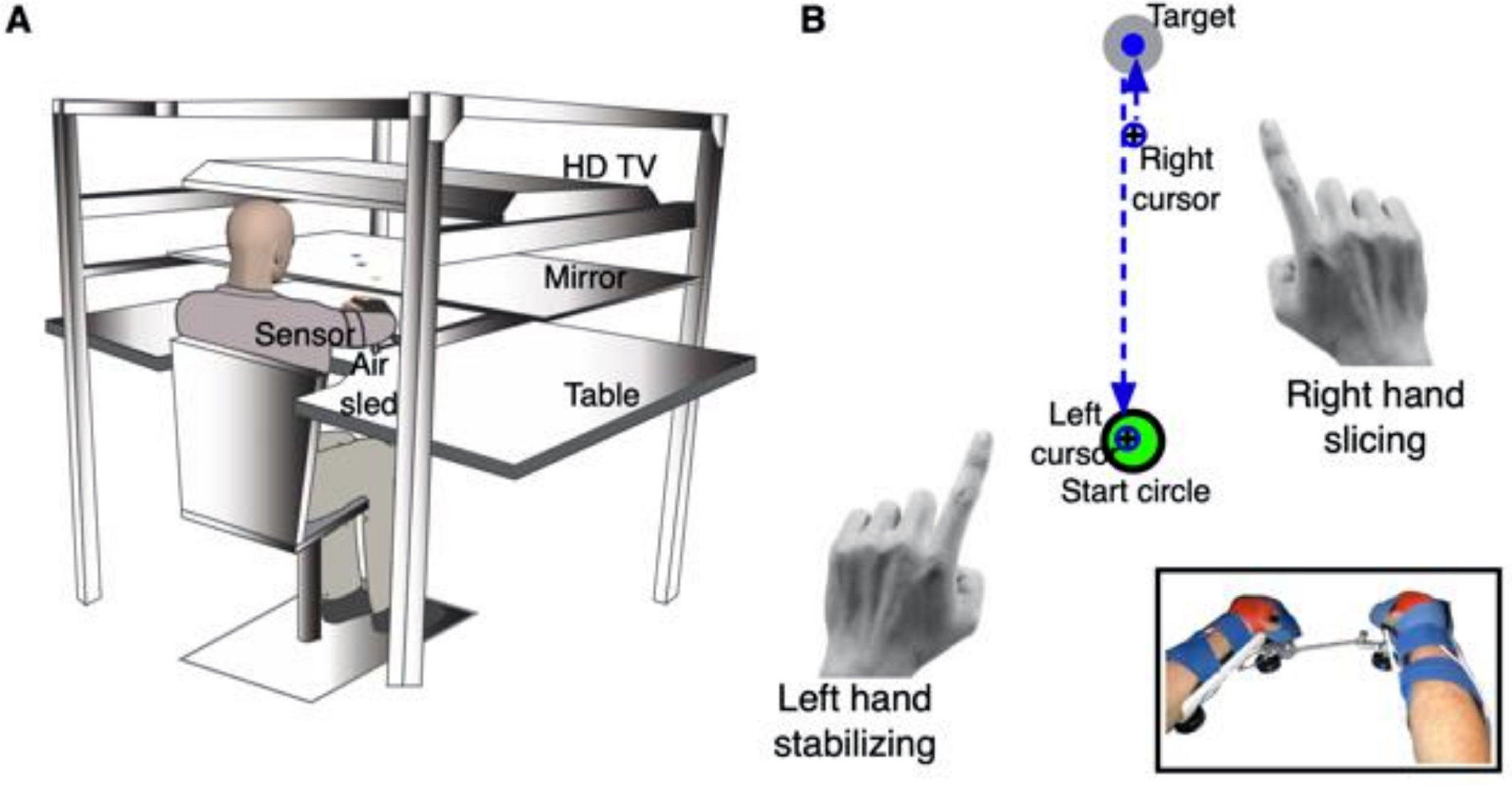
Experimental setup and task. (**A**) Participants were seated on a chair in front of the KineReach virtual reality system. The task was projected onto a mirrorized screen placed at chin level. Magnetic sensors attached to the upper arm and hand recorded limb position and orientation of each hand. (**B**) Each hand was strapped on to an air sled, with the two sleds mechanically connected via a spring (inset). A trial involved the target appearing in one of three directions and requiring the “slicing” hand to make a quick out-and back movement while the “stabilizing” hand kept the corresponding cursor inside the start circle.

We used the KineReach virtual reality motion tracking system (developed and programmed by R.L. Sainburg) to conduct the experiment (Figure 1A). A participant sits on a height-adjustable chair and rests their chin on a horizontal mirrored screen that occludes view of the hands placed on the table. An inverted HD monitor placed above the participant’s head projects the task interface onto the screen so that the task appears in the same horizontal plane as the hands. We recorded limb position and orientation of each arm at 116 Hz using two 6-degree-of-freedom magnetic sensors (trakStar^®^; Ascension Technology) placed on the upper arm and hand. We computed arm movements from digitized bony landmarks to estimate finger, wrist, elbow and shoulder joint positions, shoulder and elbow angles. All joints distal to the elbow were immobilized using an adjustable brace, and each hand was supported on an air sled that glided on the table’s glass surface to reduce the mechanical effects of friction and gravity on movement. The air sleds were connected by a spring (spring constant: 75.3 N/m) attached at the ends to produce a mechanical coupling of the hands. A cursor (1.5 cm diameter) representing each hand was visible on the screen. Each cursor was displaced 12.7 cm medially from the corresponding hand to prevent the hands from colliding at the start position. Previous research has shown that such lateral displacement of the cursor does not affect trajectory direction or movement accuracy [31].

Each participant completed the “slicing and stabilizing” task under two conditions: (1) right hand slicing and left hand stabilizing, and (2) left hand slicing and right hand stabilizing. The slicing task was chosen because previous research has shown that reversing the motion reflects trajectory control mechanisms, and does not recruit the postural mechanisms required to stop at the target [32]. The order of these conditions was counterbalanced between participants. The task consisted of 36 trials where participants needed to produce an out-and-back movement with the slicing hand while keeping the stabilizing hand in one place (Figure 1B). Each trial was initiated when both cursors were placed inside a central start circle (3 cm diameter) for 300 ms, which required a stretch of the spring connecting the two arm sleds. A target circle (3 cm diameter) appeared 20 cm from the start circle pseudorandomly in one of three directions, and in the same hemispace as the slicing hand (i.e., 0°, 45°, 90° for the right hand; 90°, 135°, 180° for the left hand). Participants were asked to use the slicing hand to reach for the target as quickly and as accurately as possible, and to then move back into the start circle while keeping the stabilizing hand’s cursor in the start circle during the entire movement. Visual feedback of each cursor (representing the slicing and stabilizing hands) was provided throughout the movement, and the complete hand path covered was provided at the end of each trial as knowledge of performance.

We used custom programs designed in IgorPro (version 6.37; WaveMetrics) to process and analyze all kinematic data. The slicing movement in each trial involved an outward movement to the target followed by an inward movement to the start circle. Movement onset was defined as the first minimum of tangential velocity that was less than 8% of the outward movement’s peak velocity. Movement offset was defined as the first minimum of tangential velocity appearing after the inward movement’s peak velocity that was less than 8% of its peak velocity. The onset and offset values from the slicing data were used to align the slicing and stabilizing time series in each trial.

We determined performance of the slicing hand using the following measures: initial direction error (a measure of accuracy of predictive control), deviation from linearity (a measure of intersegmental coordination), and movement error at reversal position (a measure of efficient trajectory control). Initial direction error was defined as the angle formed by the line connecting the start circle and the cursor’s position at peak velocity, and the line connecting the target and start circle. Deviation from linearity was determined by dividing the hand path’s minor axis by its major axis, where the major axis was the largest distance between any two points on the path, and the minor axis was the largest distance between any two points perpendicular to the major axis. Error at reversal position was defined as the distance between the cursor at the end of outward reach and the target. The end of the outward reaching movement was defined as the global minimum in tangential velocity between the outward and inward cursor movements.

We determined performance of the stabilizing hand using the following measures: maximum distance moved and peak acceleration. The maximum distance moved by the hand between movement onset and offset was its largest distance from the start of movement. These measures allowed us to examine how well the hand could stabilize against the spring load that varies with the displacement of the slicing hand.

All statistical analysis were conducted in JMP Pro (Version 14, SAS Institute, Cary, NC, USA). We used a mixed model ANOVA with group (stroke, controls) as the between-subjects variable, and hand (ipsilesional, contralesional) as the within-subject variable to test their effects on each of the performance measures. In controls, the ipsilesional hand was defined as the dominant right hand, and the contralesional hand the nondominant left hand. We modeled subject as a random effect and used a Type I error rate of 0.05. Before conducting an ANOVA, we tested for normality (the Shapiro–Wilk test) and sphericity (Mauchly’s test) in each performance measure. Violations of normality were corrected by using a log transformation of the measure. Since we predicted larger hand asymmetries in stroke survivors compared to controls, we expected our ANOVA to detect a group × hand interaction.

## 3. Results

### 3.1. Reaching Movement Deficits Are Larger in Stroke Survivors Compared to Controls

**Figure 2.**
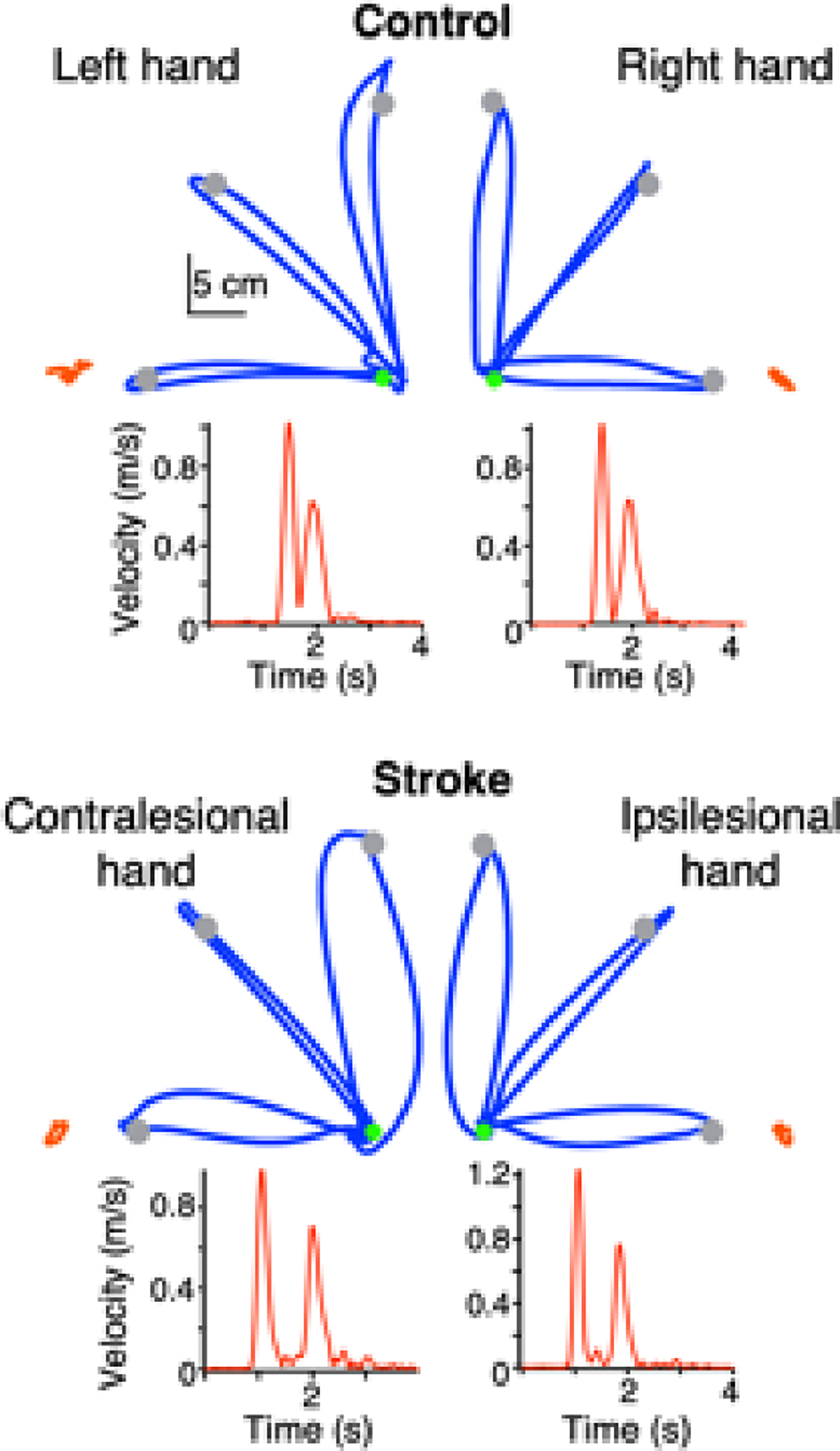
Performance during the slicing movement was impaired in stroke survivors compared to control participants. Stroke survivors produced curved out-and-back reaching movements, with target reversals that were not as sharp as those made by controls. Representative stabilizing hand trajectories for one target in each subgroup are shown in orange on the same scale as the slicing hand trajectories.

Figure 2 shows a typical slicing trajectory (hand path and tangential hand velocity profile) for a representative control participant and for a stroke participant. Control participants generally produced smooth out-and-back movements with sharp reversals, similar to results from previous work [33]. Stroke survivors, on the other hand, produced more curved movements with less sharp reversals, especially for the medial and lateral targets.

**Figure 3.**
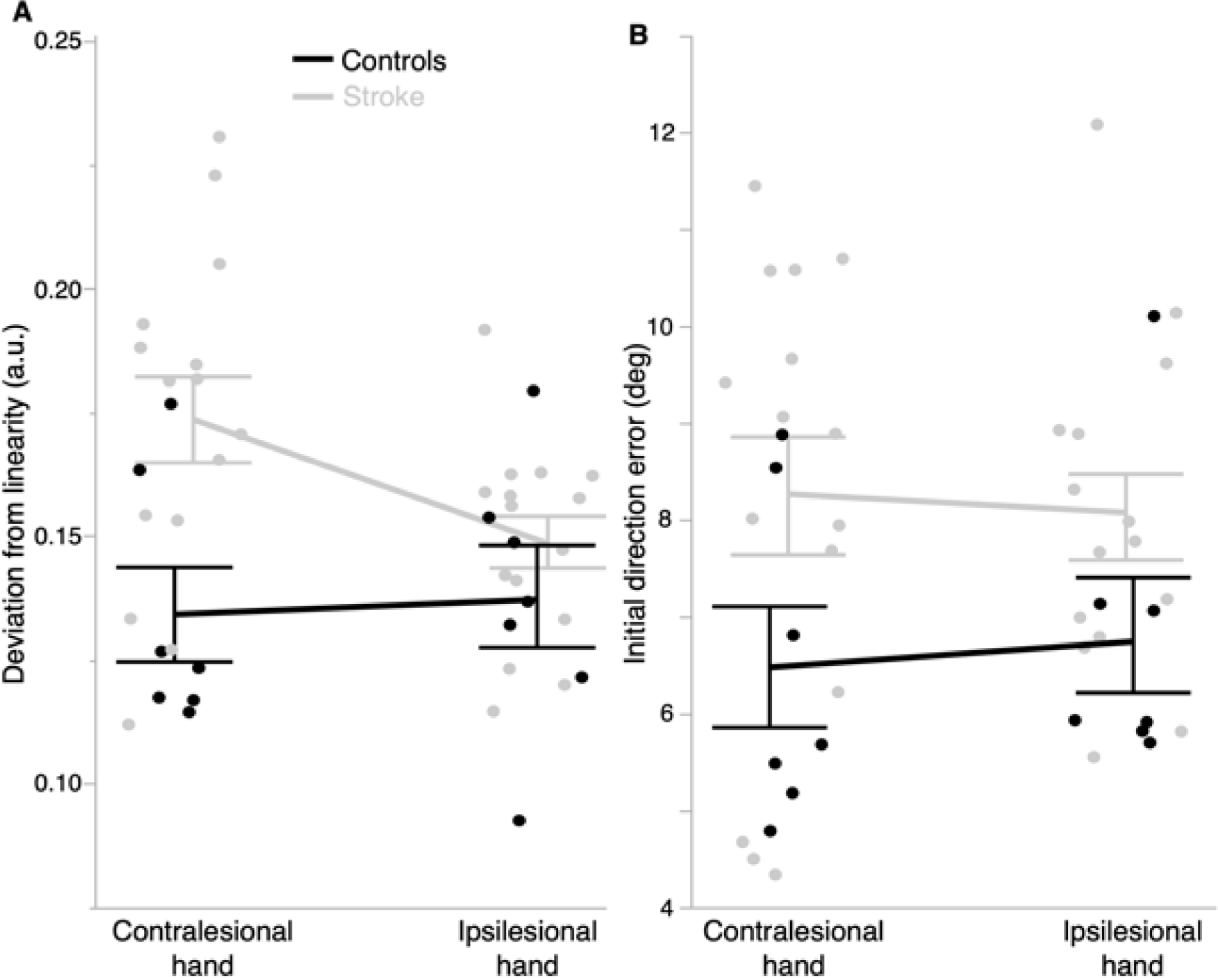
Control of reaching performance is more impaired in stroke survivors compared to controls. (**A**) Stroke survivors produced significantly less linear reaching movements compared to controls (*p* < 0.05), and their contralesional hand performance was worse than that of the ipsilesional hand. (**B**) Stroke survivors also produced significantly larger initial direction errors compared to controls (*p* < 0.05). Error bars represent 1 SEM. Each marker within a hand group represents a participant.

We examined performance between hands of stroke survivors and controls during the reaching movement (Figure 3). Our mixed model ANOVA for deviation from linearity detected a statistically significant main effect of group (*F* (1,20) = 6.57, *p* = 0.019), but not of hand (*F* (1,20) = 1.36, *p* = 0.19) or group × hand interaction (*F* (1,20) = 3.35, *p* = 0.082). Post-hoc analysis revealed significant differences between hands in stroke survivors (*p* = 0.047), with the contralesional hand producing less linear movements compared to the ipsilesional hand. Results for initial direction error indicated a statistically significant main effect of group (*F* (1,20) = 4.36, *p* = 0.0498), but not of hand (*F* (1,20) = 0.011, *p* = 0.92) or group × hand interaction (*F* (1,20) = 0.27, *p* = 0.61). In terms of efficient control of trajectory during reversal, our mixed model ANOVA for movement error at reversal position produced a statistically significant main effect of hand (*F* (1,20) = 8.45, *p* = 0.0087), but not of group (*F* (1,20) = 0.88, *p* = 0.36) or group × hand interaction (*F* (1,20) = 1.16, *p* = 0.29). The contralesional hand (which is the left hand in controls) produces more errors at reversal compared to the ipsilesional hand. Overall, our results suggest that the stroke survivors in this study produced deficits in movement planning and intersegmental coordination, regardless of which hand was used.

### 3.2. Stabilizing Behavior Is Similar between Controls and Stroke Survivors

**Figure 4.**
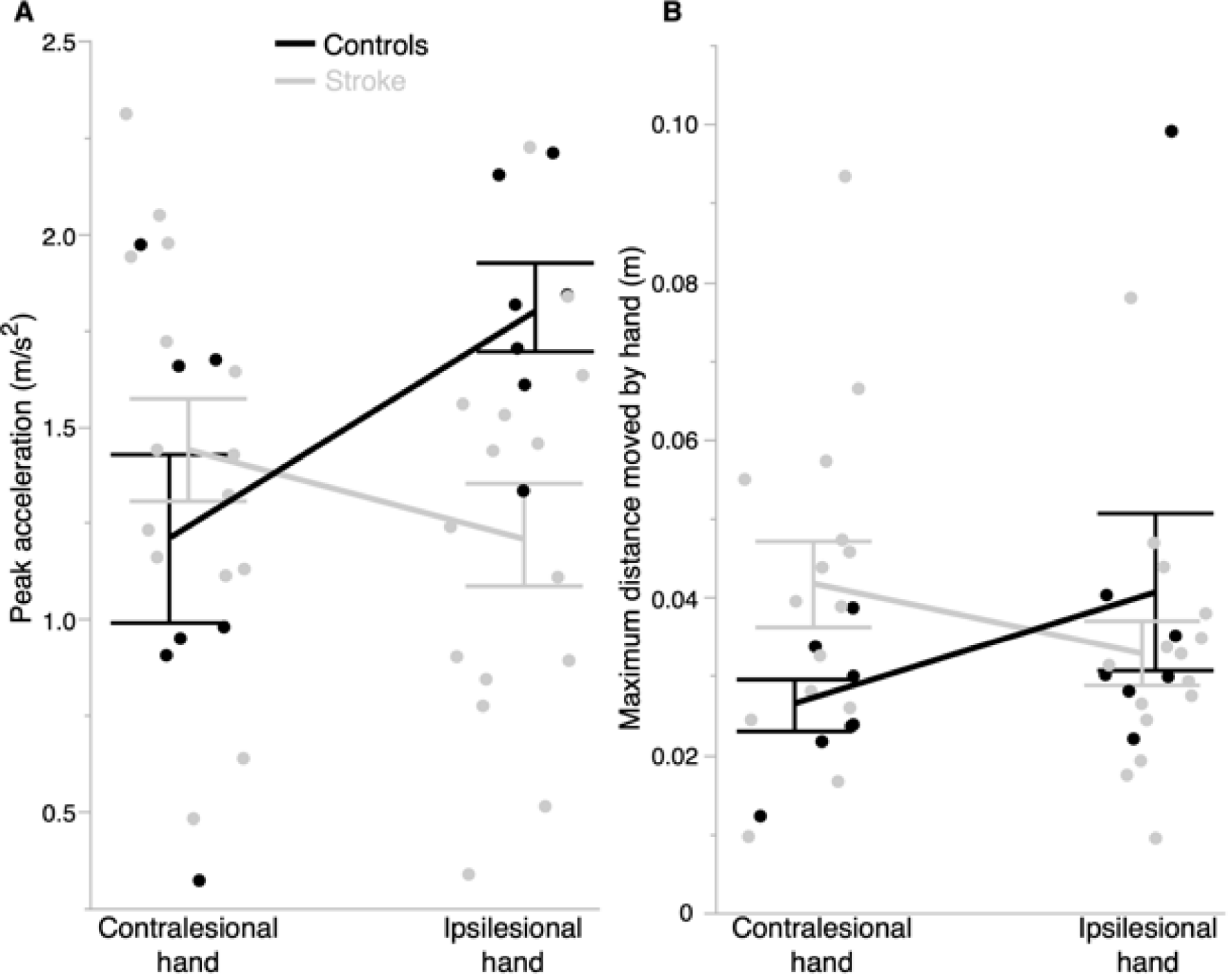
Control of steady position is similar between controls and stroke survivors on this mechanically coupled bilateral task. (**A**) There was a significant group × hand interaction effect for peak acceleration (*p* < 0.05), such that the ipsilesional and contralesional hands of stroke survivors exhibited similar stabilizing behaviors. (**B**) There were no differences between groups and hands for maximum distance moved by the stabilizing hand. Error bars represent 1 SEM. Each marker within a hand group represents a participant.

We examined differences between the two groups in the ability to maintain a steady position with the stabilizing hand while the other hand was performing the slicing movement. Figure 4 shows the average peak acceleration and maximum distance moved by the stabilizing hand for each group’s ipsilesional and contralesional hands. Our mixed model ANOVA for peak acceleration identified a statistically significant group × hand interaction (*F* (1,20) = 6.75, *p* = 0.017), such that the dominant right hand of controls accelerated more than the nondominant left hand (mean difference: 0.60 m/s^2^; *p* = 0.13). In contrast, peak acceleration of the ipsilesional and contralesional hands of stroke survivors was not different from one another (mean difference: 0.22 m/s^2^; *p* = 0.61). There were no main effects of group (*F* (1,20) = 1.19, *p* = 0.29) or hand (*F* (1,20) = 1.45, *p* = 0.24). While the spring forces imposed by motion of the reaching hand produced such interaction effects with regards to peak acceleration, these effects were not significant for the maximum distance moved by the hand (*F* (1,20) = 4.04, *p* = 0.058). There were also no main effects of group (*F* (1,20) = 0.31, *p* = 0.59) or hand (*F* (1,20) = 0.36, *p* = 0.55). Thus, our results suggest that control of postural features in such a task may produce similar behaviors between hands of stroke survivors.

## 4. Discussion

We examined asymmetries in performance of unilateral stroke survivors and neurologically intact individuals on a bilateral task that required complementary coordination between the two hands. We expected that stroke survivors would produce larger deficits in contralesional, compared to ipsilesional, hand performance on both reaching and stabilizing aspects of behavior. Our results partially supported our hypothesis since we observed reaching performance deficits in stroke survivors compared to controls, specifically in the contralesional hand. However, stabilizing performance was not different between the groups. The dominant right hand of control participants showed worse stabilizing performance than that of the nondominant left hand, a finding supported by previous literature [24,34]. In contrast, stroke survivors showed no significant differences in stabilization relative to controls, nor differences between the hands (contralesional or ipsilesional). These findings suggest that mild hemiparesis results in deficits in trajectory control while postural control may be less affected during this type of coupled bilateral task.

Our current findings indicate trajectory deficits during the slicing movement, regardless of hand used (contralesional, ipsilesional), for stroke survivors with mild paresis. Substantial previous literature has reported trajectory deficits during unilateral reaching tasks in both the ipsilesional arm of left hemisphere damaged individuals, as well as in the contralesional arm of individuals with mild paresis [18,20]. In the current study, we did not explore the role of the lesioned side on our results because the sample size would not provide sufficient power to conduct such an analysis. However, we observed more curved movements and larger initial direction errors when completing the out-and-back slicing movement in stroke survivors compared to controls. These results extend the findings of previous unilateral as well as bilateral reaching studies to a task requiring asymmetrical coordination for control of a mechanical couple between the hands. More research is required to determine whether the deficits found in this bilateral task might vary with the side of the brain that is lesioned, as demonstrated in previous unilateral reaching studies [20].

In contrast to our trajectory control findings, our results in stroke survivors indicating no deficits in stability control appear to partially contrast with previous studies of unilateral reaching. Previous research has reported deficits in achieving stable final positions during reaching in right, but not left hemisphere damaged stroke survivors [16]. Our current findings showed that both hands of stroke participants stabilized similarly well in this task, and importantly, performed better than the dominant right hand of controls. Although previous research had not examined the ability of stroke survivors to stabilize one hand against a mechanically coupled load that varies with the displacement of the other hand, we predicted that deficits in achieving final stable positions at the end of a unilateral reaching movement might require similar mechanisms of impedance control [32,35]. There are several possible explanations for our stabilization results. First, and as mentioned previously, we could not determine the role of the lesioned hemisphere on these effects. The role of right hemisphere damage in impedance control may have been diluted by the inclusion of individuals with left hemisphere damage in the overall stroke group. Second, our group of stroke survivors had mild paresis in the contralesional arm and little-to-no functional deficits in the ipsilesional arm; hence, motor deficits in both arms were minimal. However, this interpretation is contradicted by our findings for trajectory deficits that are significant for this task in both arms of stroke survivors. Nevertheless, the inclusion of only stroke survivors with mild paresis in the current study was a significant study limitation, which does not allow us to extend these findings to individuals with more substantial contralesional and ipsilesional arm deficits. We imposed this restriction to assure that all participants would be able to perform this task without restrictions associated with abnormal synergies and spasticity in the contralesional arm. Finally, it is plausible that the type of stabilizing control required in our bilateral task did not reflect the control mechanisms underlying the final position deficits reported for both arms of right hemisphere damaged individuals in previous studies [20].

In conclusion, our findings emphasize that performance during bilateral asymmetrical and mechanically coupled movements following stroke is similar to unilateral movement only during initial trajectory specification and not during the stabilizing phase. We expect that further research on the mechanisms of bilateral control is necessary to understand the impact of stroke on bilateral coordination. It is possible that increased competition for neural resources during bilateral movement may increase activity of contralateral motor pathways and/or ipsilateral corticospinal pathways, leading to the potential for compensatory movement or even recovery [2]. It is important to note, though, that bimanual movements in real-world activities can include a range of behaviors in which one arm does not always have to play a stabilizing role [34], such as when washing your hands or wrapping a gift. Importantly, stroke survivors with mild hemiparesis, such as in the group of individuals recruited for this study, are often capable of functional bimanual coordination that can be utilized in the rehabilitation strategy to improve their functional outcomes [12]. It may be possible that when remediating bilateral movements in stroke, using the ipsilesional hand for manipulation while using the contralesional hand for stabilizing objects may be an effective strategy. Future work can determine whether this strategy can be effective even when premorbid handedness is also considered.

## Figures and Tables

**Table 1.** Demographics of stroke participants. Lesion side: R (right hemisphere), L (left hemisphere); Chronicity: years post stroke; Upper extremity portion of the Fugl-Meyer score is out of 66 points; Jebsen-Taylor hand function test total score is provided in seconds with the writing score excluded.

Participant	Sex	Age	Lesion Side	Chronicity	Fugl-Meyer	Jebsen-Taylor

1	M	77	R	5.5	60	30
2	M	68	R	5.5	54	36
3	M	58	R	6	56	39
4	M	77	R	5.6	62	41
5	M	58	R	20.9	53	53
6	M	79	R	3.6	65	37
7	F	37	R	6.7	49	25
8	F	62	R	4.5	64	35
9	M	45	L	6.5	65	24
10	M	67	L	6.6	62	34
11	M	71	L	3.3	66	33
12	M	51	L	4.4	66	37
13	M	64	L	2	62	55
14	F	56	L	4	65	26
15	F	55	L	1.4	65	34.05

## Data Availability

The data presented in this study are available on request from the corresponding author.
